# Optimizing thermal radiation control with ultra-broadband metamaterials for high passive radiative cooling efficiency

**DOI:** 10.1038/s41598-025-27263-8

**Published:** 2025-12-04

**Authors:** Tesfaye Feyisa, Abebe Belay, Fekadu Tolessa, Kusse Kudishe, Umer sherefedin, Dereje Gelanu, Jebel Haji, Desta R. Golja, Manza Zityab

**Affiliations:** 1 Department of Applied Physics, School of Applied Natural Science, Adama Science and Technology University, Adama, Ethiopia; 2 Department of Applied Physics, School of Applied Natural Science, Jinka University, Jinka, Ethiopia; 3 Department of Applied Physics, School of Applied Natural and computational Science, Madda Walabu University, Bale Robe, Ethiopia

**Keywords:** Electronic structure, Metamaterial, Solar irradiation, Selective-emissivity, Atmospheric window, Radiative cooling, Climate sciences, Environmental sciences, Energy science and technology, Materials science, Optics and photonics, Electronics, photonics and device physics, Optical physics

## Abstract

Managing high energy consumption and thermal energy has become crucial for ensuring a sustainable and stable environment. Recently, passive radiative cooling (PRC) has emerged as an innovative method for reducing environmental energy density without requiring external energy input. This study focused on three wavelength ranges: 2.5–5 μm, 8–13 μm, and 16–27 μm, to optimize net cooling power. We acquired the optical and electrical properties of the materials utilized in this study through density functional theory (DFT). A honeycomb structure was designed as a spectrally selective emitter by using Finite Element Method (FEM) method to enhance radiative properties. We analyzed how geometric parameters affect absorbance and emissivity performance. With the optimal geometry, we achieved a net cooling power of 150.4 W/m² under 994 W/m² of direct solar irradiation during the day. At night, in the absence of sunlight, the net cooling power increased to 198 W/m². The system reached equilibrium temperatures of 256 K during the day and 244 K at night, assuming an ambient temperature of 300 K. Even when considering parasitic convection and conduction, the cooler successfully maintained sub-ambient temperatures. Furthermore, the designed cooler exhibited polarization independence and high emissivity across a wide range of incidence angles (from 0° to 75°).

## Introduction

The increasing dependence on fossil fuels has resulted in a rise in greenhouse gas emissions, which contributes to the ongoing increase in Earth’s temperature^1^. Consequently, cooling technology has become a crucial requirement for everyday life and industrial processes^2,3^. Active cooling systems have been implemented to tackle challenges related to space cooling; however, they present their own issues by necessitating a constant energy supply^4,5^. These factors lead to environmental instability and threaten energy security. To reduce the consumption of nonrenewable energy and safeguard the environment, various strategies have been developed^6,6–8^. Among these, passive radiative cooling (PRC) has garnered attention for its ability to transfer heat from the Earth’s surface to the cold outer space (3 K) through the atmospheric transparency window, all without needing external energy input^9,10^. PRC is notable for its lack of harmful gas emissions, its capacity to minimize energy waste, and its classification as a renewable energy source^11^.

The Earth’s atmosphere exhibits three infrared transparency windows: near-infrared (NIR) spanning 2.5 μm to 5 μm, mid-infrared (MIR) ranging from 8 μm to 14 μm, and far-infrared (FIR) covering 16 μm to 27 μm^12,13^. By leveraging these windows to radiate heat from the Earth’s surface into outer space (3 K) while reflecting solar radiation (0.3 μm to 2.5 μm), it becomes possible to cool environments or materials below ambient temperatures without relying on external energy input^14^. The passive cooling system is significantly influenced by solar radiation and can be broadly categorized into two types: nighttime^15^ and daytime cooling^16^. Early research efforts primarily focused on achieving passive radiative cooling at night, whereas daytime cooling remained challenging due to the dominant influence of solar radiation. For instance, Hossain et al.^17^ developed a highly efficient nighttime cooling system using a selective emitter based on an Al/Ge structure, while Chen et al.^18^ explored experimental and theoretical approaches involving polymer coatings. However, these methods were only effective during nighttime and required the incorporation of a solar reflector.

Numerous efforts have been made to develop an optimal solar reflector and thermal emitter simultaneously^19^. The traditional method for achieving daytime cooling relies on solar reflectors such as ZnS, ZnSe, and polymer broadband emitters^20^. However, these materials have a solar reflectance of less than 85%, which limits their ability to cool below ambient temperatures during the day. Thus, it is crucial to optimize solar reflectance while also enhancing thermal emission to design an effective daytime radiative cooler. In line with this principle, several researchers have created daytime cooling systems. Rephaeli et al.^20^ conducted the first numerical study on photonic materials that effectively reflect solar radiation (0.3–2.5 μm), while Raman et al. demonstrated a single planar device with a solar reflectance of 97% through experimental means. Despite achieving high solar reflection, their designs compromised thermal emittance and added complexity, which ultimately decreased overall cooling efficiency. More recently, Chen et al.^21^ proposed mesoporous photonic structures and random dielectric microsphere coatings that show excellent solar reflectance and strong mid-infrared emittance^24^. However, both studies reported a net cooling power of less than 80 W/m².

Recently, various material structures have become increasingly popular in experimental and numerical research to improve cooling efficiency during both day and night. These include photonic structures^21,22^, porous and coating polymers^18,23^, nanoparticles^24^, plasmonic and microsphere coatings^25,26^, and Metal-Dielectric-Metal (MDM) metamaterials^17,27^. Thin-film optical filters based on MDM structures, in particular, have garnered considerable interest^28^. For example, Lee et al. (2017) and Liu et al. (2021) explored MDM designs with one-window and narrow-band absorber/emitter configurations for nighttime applications^27^. In 2022, Zu et al.^29^. conducted experimental studies using zeolite, a material known for its high solar reflectivity. Furthermore, in 2023, Yan et al.^30^. developed two complex 10-layer SiO₂ metamaterial radiative coolers incorporating cylindrical MgF₂ doping. However, achieving both unity emissivity in the infrared atmospheric windows and high solar reflectance remains a significant challenge requiring substantial advancements^31^. MDM structures, as artificial materials exhibiting unique properties not found in nature, have attracted attention for their potential to enhance cooling performance. Veselago^32^ first conducted theoretical studies on electromagnetic MDM structures in 1968, and Pendry et al.^33^ provided the first experimental validation in 2000.

Creating a radiative cooler that combines strong solar reflectivity with near-ideal, highly selective infrared emissivity is a major challenge, especially for daytime cooling. In this regard, Chongjia Lin et al.^34^ studied structures with high solar reflectance and high thermal emissivity in the 8–13 μm range. However, they focused mainly on a single window, 8–13 μm. Therefore, their cooling performance below ambient temperature is minimal compared to our work. Furthermore, many prior studies have not adequately considered conduction and convection losses (P_(cond+conv)_) in their net power calculations, leading to an overestimation of cooling efficiency. Our study overcomes all these challenges by focusing on three atmospheric windows and considering conduction-convection power, which face 994 W/m² units of solar power but results in a high total net cooling. This research seeks to maximize net cooling power and achieve sub-ambient cooling by leveraging high solar reflectance and near-unity thermal emittance across three spectral windows using an MDM structure. To overcome these limitations, we introduce a new five-layer MDM micro-honeycomb design capable of cooling below ambient temperature, even when factoring in conduction and convection losses. Our results demonstrate a significant net cooling power of 150.4 W/m² during the day and 198 W/m² at night. Compared to earlier research, this study presents improvements in achieving high infrared absorption/emission across all three windows, superior solar reflection, increased net cooling power, use of cost-effective materials, and simplified fabrication.

## Materials and numerical methods

### Materials

In this study Ni and TiO2 materials used in computational and experimental are considered. DFT calculations were performed using Quantum ESPRESSO and Materials Studio to get electronic and optical properties of TiO₂ and Ni. They were chosen because of their high dielectric constants, excellent thermal stability, low optical losses, high emissivity for thermal radiation, and insulating properties. The PAW method with Quantum ESPRESSO pseudopotentials described core-valence interactions. The electronic and optical properties of TiO₂ and Ni, determining band structure, DOS, and refractive index via the dielectric function were studied. CIFs were sourced from the Materials Project, and the crystal structure was optimized using the PBE-GGA approach.

### Geometry

**Fig. 1:**
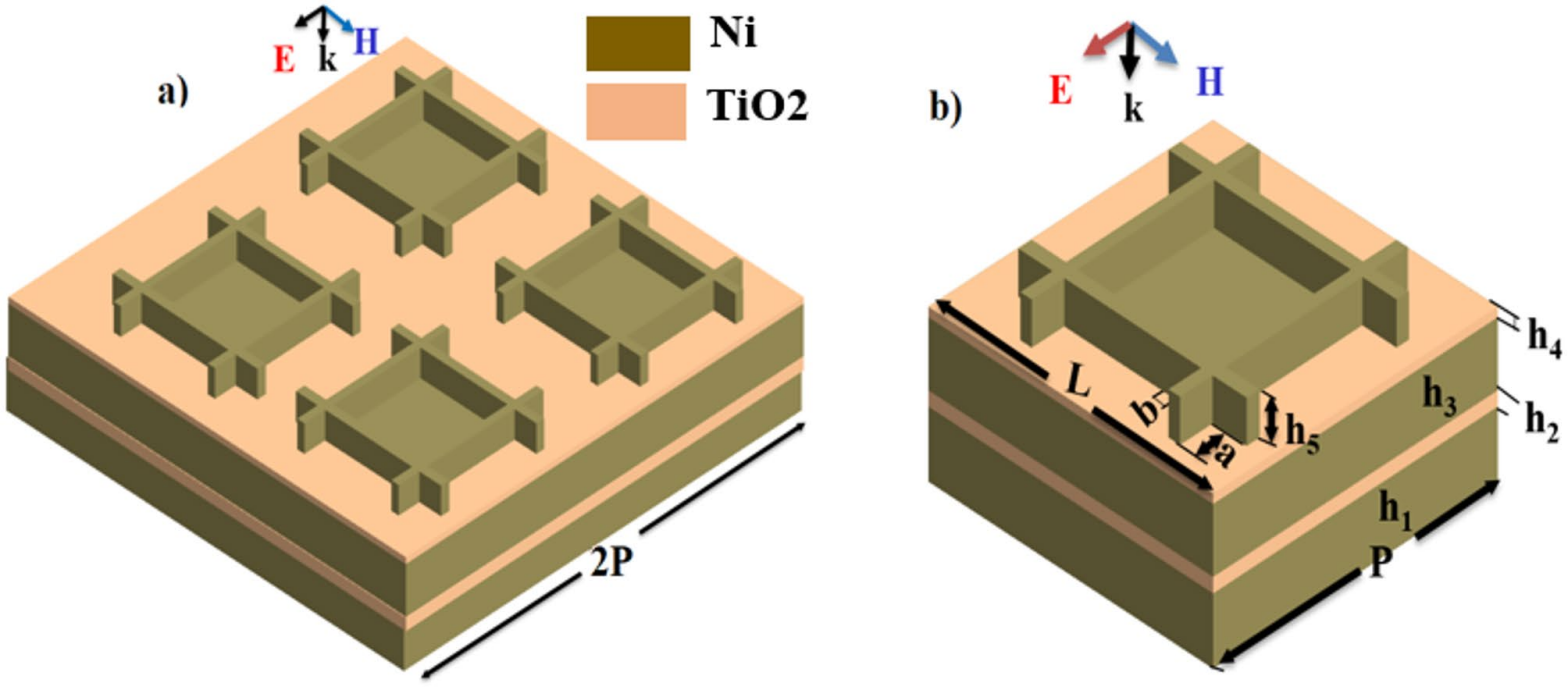
(**a**) Three dimensions (3D) honeycomb MDM for passive radiative cooling model. (**b**) The plane of incidence and polarization.

A variety of computational packages and software tools were employed for simulations, numerical calculations, data visualization, and graphical plotting. For computational calculations such as electronic properties, band gaps, and dielectric functions of materials, the QUANTUM ESPRESSO package and Materials Studio were utilized. The 3D honeycomb MDM absorber/emitter is designed using a Ni film and a TiO₂ spacer, as illustrated in Fig. 1(a). In this configuration, the optical properties of TiO₂ and Ni are determined using Density Functional Theory (DFT). A 1 W plane wave is incident along the z-axis, with periodic boundary conditions applied in the x and y directions. Numerical analysis utilizing the Finite Element Method (FEM) in COMSOL-MULTIPHYSICS encompassed structural design and parameter evaluation, including a perfectly matched layer to minimize reflections^35^. The emitter produces magnetic polarization (MP) and surface plasmon polaritons (SSP), which enhance absorptivity and emissivity within the target wavelength range. These properties were evaluated using Kirchhoff’s law, and fabrication can be achieved through e-beam patterning or plasma etching^36,37^.

For visualization and plotting of results, several software applications were implemented, including VESTA for electronic and structural analysis, XCrysDen for crystalline structures and densities, and XMGrace, along with Origin Pro 2022 for diffraction analysis and image processing. Finally, Microsoft Excel was used to integrate and implement the data. These technologies enhanced the overall clarity and effectiveness of the research by making it easier to convert complex data into visually intelligible representations. At the end, the effective execution of the computations, data analysis, and presentation of the findings were validated.

The directional emittance can be derived using Kirchhoff’s law of thermal radiation by calculating the directional absorbance of incident light on the grating structure, as illustrated in Fig. 1b. Relative to the plane of incidence, the incident wave vector is characterized by polar angles (θ), azimuthal angles (ɸ), and polarization angles (ψ), assuming the radiation is linearly polarized. Here, ψ denotes the angle between the electric field E and the plane of incidence, while ɸ represents the angle between the x-z plane and the plane of incidence, which encompasses both the incident light and the z-axis. The propagating wave that is the incident plane wave has the wave vector $\:{\mathbf{K}}_{\text{i}\text{n}\text{c}\text{d}}=({\text{K}}_{\text{x},\:\:\text{i}\text{n}\text{c}\text{d}},\:{\text{K}}_{\text{y},\:\text{i}\text{n}\text{c}\text{d}},\:\:{\text{K}}_{\text{z},\:\text{i}\text{n}\text{c}\text{d}})$ which is given by;

1 $$\:{\text{K}}_{\text{x},\:\:\text{i}\text{n}\text{c}\text{d}}={\text{k}}_{\text{o}}{\text{n}}_{\text{a}\text{i}\text{r}}\text{s}\text{i}\text{n}{\uptheta\:}\text{c}\text{o}\text{s}\Phi,\:{\text{K}}_{\text{y},\:\:\text{i}\text{n}\text{c}\text{d}}={\text{k}}_{\text{o}}{\text{n}}_{\text{a}\text{i}\text{r}}\text{s}\text{i}\text{n}{\uptheta\:}\text{s}\text{i}\text{n}\Phi,\:{\text{K}}_{\text{z},\:\:\text{i}\text{n}\text{c}\text{d}}={\text{k}}_{\text{o}}{\text{n}}_{\text{a}\text{i}\text{r}}\text{c}\text{o}\text{s}{\uptheta\:}$$

Where; $\:{\text{n}}_{\text{a}\text{i}\text{r}}$ is refractive index of air, indicate the source of radiation through air and the free space wave vector is defined as $\:{\text{K}}_{0}=\frac{2\pi\:}{{{\uplambda\:}}_{o}}$, where $\:{{\uplambda\:}}_{o}$ is the wavelength in vacuum. Any incident wave with a polarization angle ψ can be decomposed into transverse magnetic (TM) waves, where ψ = 0°, and transverse electric (TE) waves, where ψ = 90°, as these two polarizations form the fundamental components of all other polarizations.

### Numerical calculations

In a material, an electromagnetic wave can undergo absorption, reflection, or transmission. The principles of energy conservation (A + T + *R* = 1) and Kirchhoff’s law, which states that emissivity equals absorptivity in thermal equilibrium, facilitate the determination of absorbance using scattering parameters (S-parameters) in simulation studies^38^. The wave’s behavior is assessed using Kirchhoff’s rule in Eq. (1).

2 $$\:{\upalpha\:}\left({\upomega\:}\right)=1-{\left|{\text{S}}_{11}\right|}^{2}-{\left|{\text{S}}_{12}\right|}^{2}$$.

Absorptivity, transmissivity, and reflectivity are represented by α(ω), S12, and S11, respectively. Given that nickel, a metal, constitutes the ground layer and opaque materials obstruct sunlight^39^, Kirchhoff’s law in Eq. (2) is refined as follows:

3 $$\:{\upalpha\:}\left({\upomega\:}\right)=1-{\left|{\text{S}}_{11}\right|}^{2}$$

The study examines a working wavelength range from 0.3 μm to 27 μm, with a unit cell period of *P* = 16 μm to ensure effective microstructure design. To evaluate the performance of the designed emitter, the average thermal emittance for three atmospheric windows ($\:{\overline{\epsilon\:}}_{ther}=2.5\:{\upmu\:}\text{m}-5\:{\upmu\:}\text{m}$,$\:\:8\:{\upmu\:}\text{m}-\:13\:{\upmu\:}\text{m}\:\text{a}\text{n}\text{d}\:16\:{\upmu\:}\text{m}-\:27\:{\upmu\:}\text{m})$ was calculated using Eq. (5) for an optimized structure. Additionally, the mean solar reflectance ($\:{\overline{\text{R}}}_{\text{s}\text{o}\text{l}\text{a}\text{r}}=0.3\:{\upmu\:}\text{m}-2.5\:{\upmu\:}\text{m}\:$) was determined by calculating the ratio of reflected solar intensity across the entire solar spectrum to the integral solar intensity within the same range, as outlined in Eq. (4).

4 $$\:{\overline{\text{R}}}_{\text{s}\text{o}\text{l}\text{a}\text{r}}=\underset{{\lambda\:}_{1}}{\overset{{\lambda\:}_{2}}{\int\:}}\text{d}{\uplambda\:}{\text{I}}_{\text{A}\text{M}1.5}({\uplambda\:},0)\text{R}({\uplambda\:},0)/\underset{{\lambda\:}_{1}}{\overset{{\lambda\:}_{2}}{\int\:}}\text{d}{\uplambda\:}{\text{I}}_{\text{A}\text{M}1.5}({\uplambda\:},0)$$

5 $$\:{\overline{\epsilon\:}}_{ther}=\underset{{\lambda\:}_{1}}{\overset{{\lambda\:}_{2}}{\int\:}}\text{d}{\uplambda\:}{\text{I}}_{\text{B}\text{B}}({\text{T}}_{\text{s}\text{u}\text{r}},{\uplambda\:}){{\upepsilon\:}}_{\text{s}\text{u}\text{r}}({\uplambda\:},{\uptheta\:})/\underset{{\lambda\:}_{1}}{\overset{{\lambda\:}_{2}}{\int\:}}\text{d}{\uplambda\:}{\text{I}}_{\text{B}\text{B}}({\text{T}}_{\text{s}\text{u}\text{r}},{\uplambda\:})$$

Here, $\:{\text{I}}_{\text{B}\text{B}}\left(\text{T},{\uplambda\:}\right)=2\text{c}\text{h}/{{\uplambda\:}}^{5}\left({\text{e}}^{\frac{\text{h}\text{c}}{{\uplambda\:}{\text{K}}_{\text{B}}\text{T}}-1}\right)$ is spectral distribution intensity of a black body according planks law at operations temperature T, λ is the wavelength, cis the speed of light, Kis the Boltzmann constant, and h is the Planck constant.

In this context, $\:{{\upepsilon\:}}_{\text{s}\text{u}\text{r}}({\uplambda\:},\:{\uptheta\:})$ indicates the directional surface emissivity of the emitter at wavelength λ, while $\:{{\upepsilon\:}}_{\text{a}\text{t}\text{m}}({\uplambda\:},{\uptheta\:})$ refers to the emissivity of the atmosphere. The combined heat transfer coefficient from conduction and convection is denoted by hc. The atmospheric spectral emissivity is defined by the equation$\:{{\upepsilon\:}}_{\text{a}\text{t}\text{m}}\left({\uplambda\:},\:\:{\uptheta\:}\right)=1-\text{t}{\left({\uplambda\:}\right)}^{1/\text{c}\text{o}\text{s}{\uptheta\:}}$, where t(λ) represents the spectral transmittance at the zenith, obtained using MODTRAN (V 6.0, Spectral Sciences Inc., USA, and Gemini Telescopes, Science and Technologies). The data is generated under the assumption of an air mass of AM1.5 and a water vapor column of 1.0 mm. It is assumed that the MDM structure is oriented toward the sun, with IAM1.5 (λ) representing the spectrum of direct normal irradiance from the sun at wavelength λ, alongside the Global Tilt spectrum with an irradiance of 994 W/m².

### Cooling power analysis

**Fig. 2:**
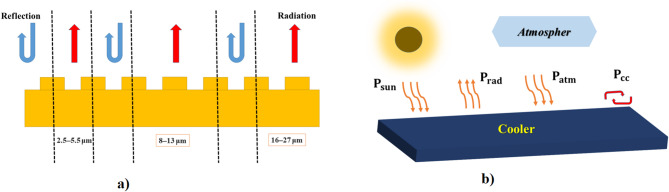
Schematic of a radiative cooling surface with four heat exchange processes.

The main factors that determine the performance of passive radiative cooling are the models and computational analyses. Two critical parameters affect cooling capacity. The first parameter is the maximization of net cooling power ($\:{\text{P}}_{\text{n}\text{e}\text{t}}$). To achieve $\:{\text{P}}_{\text{n}\text{e}\text{t}}>\:0$ for a specific surface temperature ($\:{\text{T}}_{\text{s}\text{u}\text{r}}$) and ambient temperature ($\:{\text{T}}_{\text{a}\text{m}\text{b}}$), emissivity must be high within atmospheric transparency windows ($\:2.5\:{\upmu\:}\text{m}-5\:{\upmu\:}\text{m},\:\:8\:{\upmu\:}\text{m}\:-13\:{\upmu\:}\text{m},\:\text{a}\text{n}\text{d}\:16\:{\upmu\:}\text{m}\:-27\:{\upmu\:}\text{m}$), along with high solar reflectivity ($\:0.3-2.5\:{\upmu\:}\text{m}$). Various factors influence net cooling power, including the emitter surface temperature ($\:{\text{T}}_{\text{s}\text{u}\text{r}}$), the surrounding air temperature ($\:{\text{T}}_{\text{a}\text{t}\text{m}}$), the emissivity of the emitter ($\:{{\upepsilon\:}}_{\text{s}\text{u}\text{r}}({\uplambda\:},\:{\uptheta\:})$, and the atmospheric emissivity ($\:{{\upepsilon\:}}_{\text{s}\text{u}\text{r}}({\uplambda\:},\:{\uptheta\:})$ as illustrated in Fig. 2. $\:{\text{T}}_{\text{s}\text{u}\text{r}}$ is closely linked to the design of the emitter^39^. The second important factor is the ambient temperature, which establishes the conditions necessary for maintaining a surface temperature lower than that of the ambient environment.

Therefore, the net cooling power of a surface ($\:{\text{P}}_{\text{n}\text{e}\text{t}}\left(\:{\text{T}}_{\text{s}\text{u}\text{r}}\:,{\text{T}}_{\text{a}\text{t}\text{m}}\right)$ is determined by comparing the power entering and leaving the surface^40^ for a given area (A), as calculated using Eq. (6).

6 $$\:{\text{P}}_{\text{n}\text{e}\text{t}}\left(\:{\text{T}}_{\text{s}\text{u}\text{r}}\:,{\text{T}}_{\text{a}\text{t}\text{m}}\right)={\text{P}}_{\text{r}\text{a}\text{d}}\left({\text{T}}_{\text{s}\text{u}\text{r}}\right)-{\text{P}}_{\text{a}\text{t}\text{m}}\left({\text{T}}_{\text{a}\text{t}\text{m}}\right)-{\text{P}}_{\text{s}\text{u}\text{n}}\left({\text{T}}_{\text{s}\text{u}\text{n}}\right)-{\text{P}}_{\text{c}\text{c}}$$

The term $\:{\text{P}}_{\text{r}\text{a}\text{d}}\left({\text{T}}_{\text{s}\text{u}\text{r}}\right)$, represents the power emitted by the proposed radiative coolers, $\:{\text{P}}_{\text{a}\text{t}\text{m}}\left({\text{T}}_{\text{a}\text{t}\text{m}}\right)$ denotes the heat received from the atmospheric thermal radiation, $\:{\text{P}}_{\text{s}\text{u}\text{n}}\left({\text{T}}_{\text{s}\text{u}\text{n}}\right)$ refers to the absorption of incident solar energy by the structure, and $\:{\text{P}}_{\text{c}\text{o}\text{n}\text{d}+\text{c}\text{o}\text{n}\text{v}}$ accounts for the power loss due to conduction and convection^20,40^. These quantities can be calculated using Eqs. (7), (8), (9), and (10), respectively.

7 $$\:{\text{P}}_{\text{r}\text{a}\text{d}}\left({\text{T}}_{\text{s}\text{u}\text{r}}\right)=2{\uppi\:}\text{A}{\int\:}_{0}^{\raisebox{1ex}{${\uppi\:}$}\!\left/\:\!\raisebox{-1ex}{$2$}\right.}\text{d}{\uptheta\:}\text{s}\text{i}\text{n}{\uptheta\:}\text{c}\text{o}\text{s}{\uptheta\:}{\int\:}_{0}^{{\infty\:}}\text{d}{\uplambda\:}{\text{I}}_{\text{B}\text{B}}({\text{T}}_{\text{s}\text{u}\text{r}},{\uplambda\:}){{\upepsilon\:}}_{\text{s}\text{u}\text{r}}({\uplambda\:},{\uptheta\:})$$

8 $$\:{\text{P}}_{\text{a}\text{t}\text{m}}\left({\text{T}}_{\text{a}\text{t}\text{m}}\right)=2{\uppi\:}\text{A}{\int\:}_{0}^{\raisebox{1ex}{${\uppi\:}$}\!\left/\:\!\raisebox{-1ex}{$2$}\right.}\text{d}{\uptheta\:}\text{s}\text{i}\text{n}{\uptheta\:}\text{c}\text{o}\text{s}{\uptheta\:}{\int\:}_{0}^{{\infty\:}}\text{d}{\uplambda\:}{\text{I}}_{\text{B}\text{B}}({\text{T}}_{\text{a}\text{t}\text{m}},{\uplambda\:}){\upepsilon\:}({\uplambda\:},{\uptheta\:}){{\upepsilon\:}}_{\text{a}\text{t}\text{m}}({\uplambda\:},{\uptheta\:})$$

9 $$\:{\text{P}}_{\text{s}\text{u}\text{n}}\left({\text{T}}_{\text{s}\text{u}\text{n}}\right)=\text{A}{\int\:}_{0}^{{\infty\:}}\text{d}{\uplambda\:}{\text{I}}_{\text{A}\text{M}1.5}({\uplambda\:},0){{\upepsilon\:}}_{\text{s}\text{u}\text{r}}({\uplambda\:},0)$$

10 $$\:{\text{P}}_{\text{c}\text{o}\text{n}\text{d}+\text{c}\text{o}\text{n}\text{v}}({\text{T}}_{\text{s}\text{u}\text{r}},\:{\text{T}}_{\text{a}\text{t}\text{m}})=\text{A}{\text{h}}_{\text{c}}({\text{T}}_{\text{a}\text{t}\text{m}}-{\text{T}}_{\text{s}\text{u}\text{r}})$$

## Results and discussions

### Crystal structure of TiO2 and Ni

**Fig. 3:**
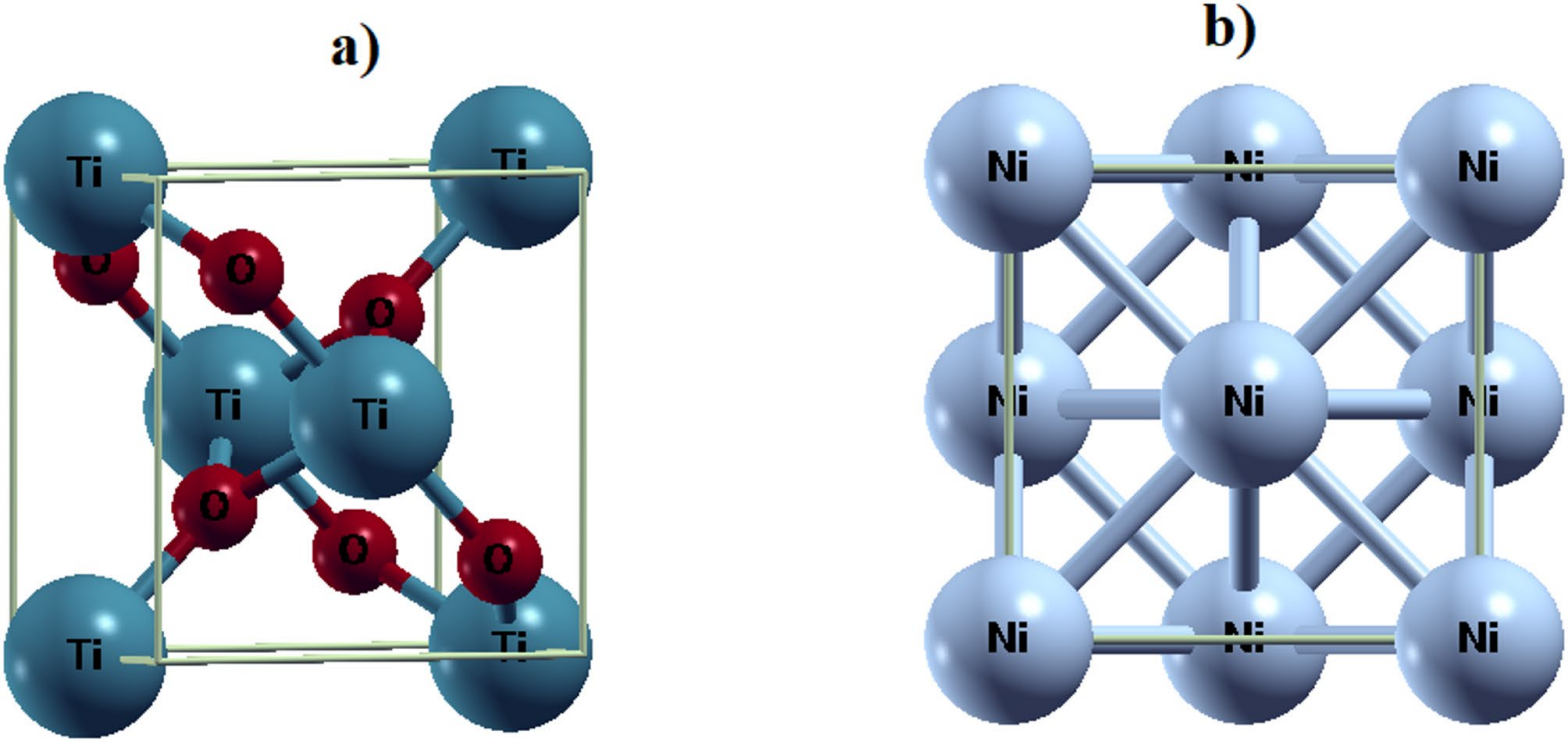
Optimize position crystal structure of; (a) TiO2. (b) Ni.

The crystal structure of anatase TiO2 is characterized by interlocking [TiO6] octahedral blocks, resulting in a three-dimensional arrangement. Within this structure, Ti-O bonds are established through strong covalent and ionic bond interactions, while the Ni has cubic (FCC) crystal structure at room temperature and connected via metallic bond. The optimized crystal structure of TiO2 and Ni are shown in Fig. 3. In this study, the optimized crystal structure of TiO2 is identified as a tetragonal structure, classified under space group 141 (I41/amd) and point group 4/m. Similar for Ni, it’s classified under space group 225 (Fm-3 m) and point group m-3 m.The calculated lattice parameters for the unit cell of each TiO2 and Ni crystal are presented in electronic band structure. This investigation contributes critical knowledge to the understanding of the electronic and optical properties of TiO2 and Ni, which is essential for different applications such as selective thermal emitter.

### Electronic band structure and density of States

**Fig. 4:**
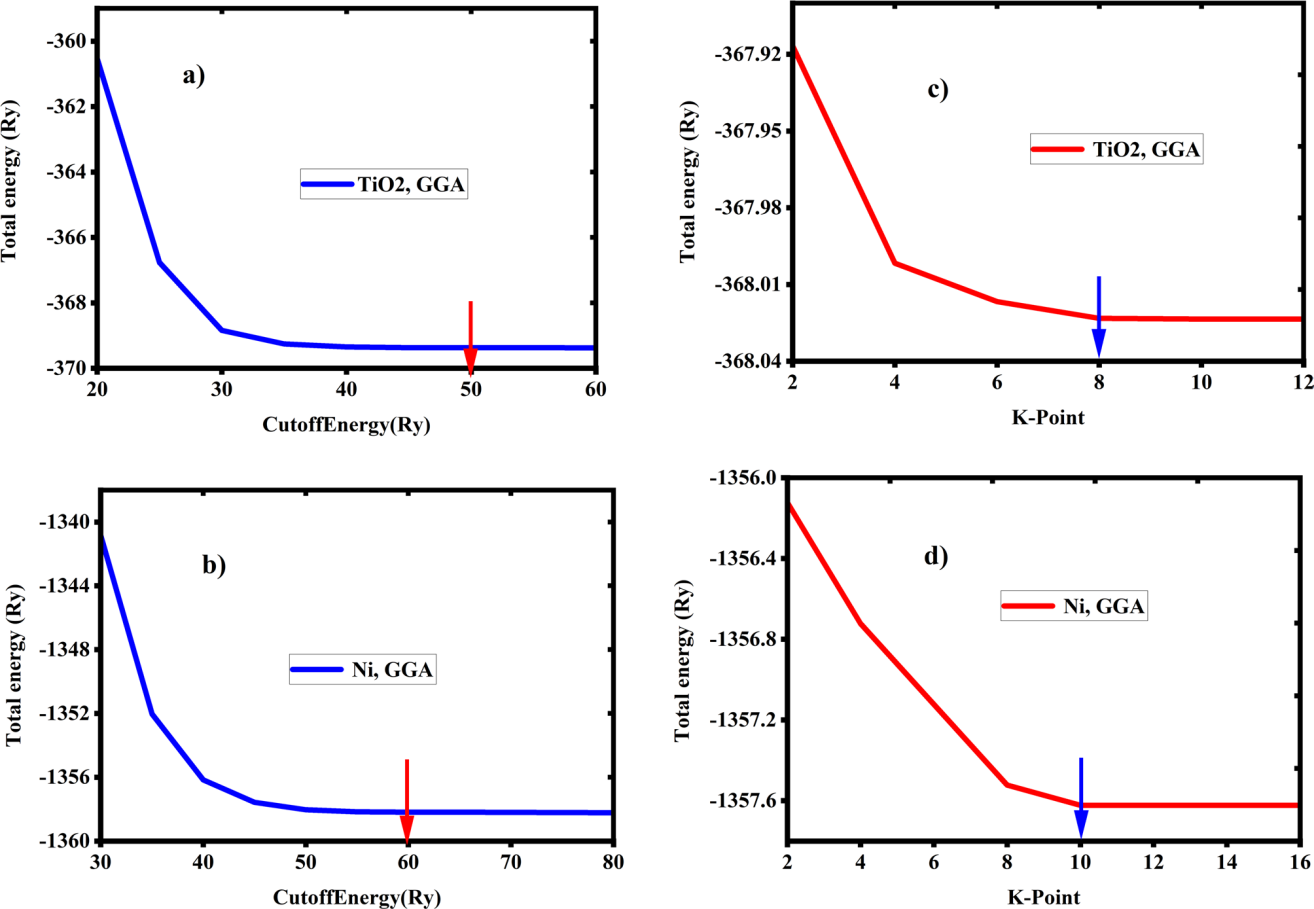
Convergence test of total energy versus cutoff energy and k-point using GGA approximations.

The electronic band structure and the density of states (DOS) are important ideas that aid in the explanation of electrical characteristics. Before analyzing the electronic structure of the system, structural optimizations were performed for the required parameters. Based on the convergence test, we selected a mesh point (7 × 7 × 7) and (6 × 6 × 6) in the first irreducible. Brillouin zone of the structure TiO2 and Ni, respectively. In addition, using the Broyden Fletcher-Goldfarb-shanno (BFGS) technique, variable cell relaxation is used to drive the minimal ground state energy littice parameter and atomic positions. The density of state calculated using (8 × 8 × 8) and (10 × 10 × 10) for TiO2 and Ni, respectively; as shown on Fig. 4. The high-symmetric K-point was employed for convenience in band-structure computations. 188 k-point was used in the irreducible Brillouin zone to calculate the band using the Monkhorst-Pack special K-point approach. The cutoff energy for a plane wave basis is 50 Ry and and 60 Ry were considered for TiO2 and Ni-respectively as shown in Fig. 4. The band gap is a crucial parameter in studying the optical properties. In this section, the electronic band structures of TiO2 and Ni crystal species were calculated.

**Fig. 5:**
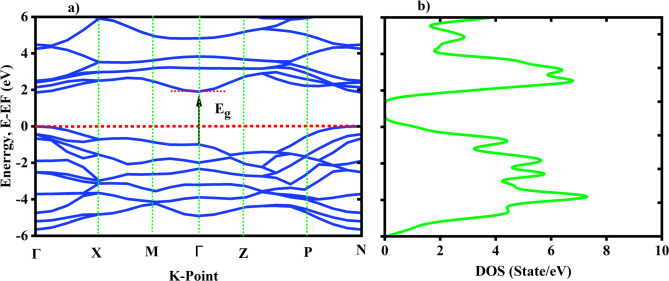
**a**) The electronic band structures of TiO2. **b**) DOS of TiO2.

The electronic band structure of the TiO2 crystal as shown in Fig. 5. From the electronic band structure, the locations of the maximum valence band (MVB) and the minimum conduction band (MCB) for TiO2 are located at different high symmetry k-points, indicating that TiO2 crystals exhibit indirect band gaps. This finding is consistent with literature report^41^. The energy band gap for the TiO2 was estimated using the exchange correlation functions of PBE and HSE06. For anatase TiO2 the band gap calculated by PBE is 2.9 eV and. This result is below the experimental results because of the approximation used by the PBE functional is underestimation. However, the energy band gaps using HSE06 functional are 3.3 eV. The result obtained from HSE06 functional is in good agreement with previously reported experimental data with^42^.

**Fig. 6:**
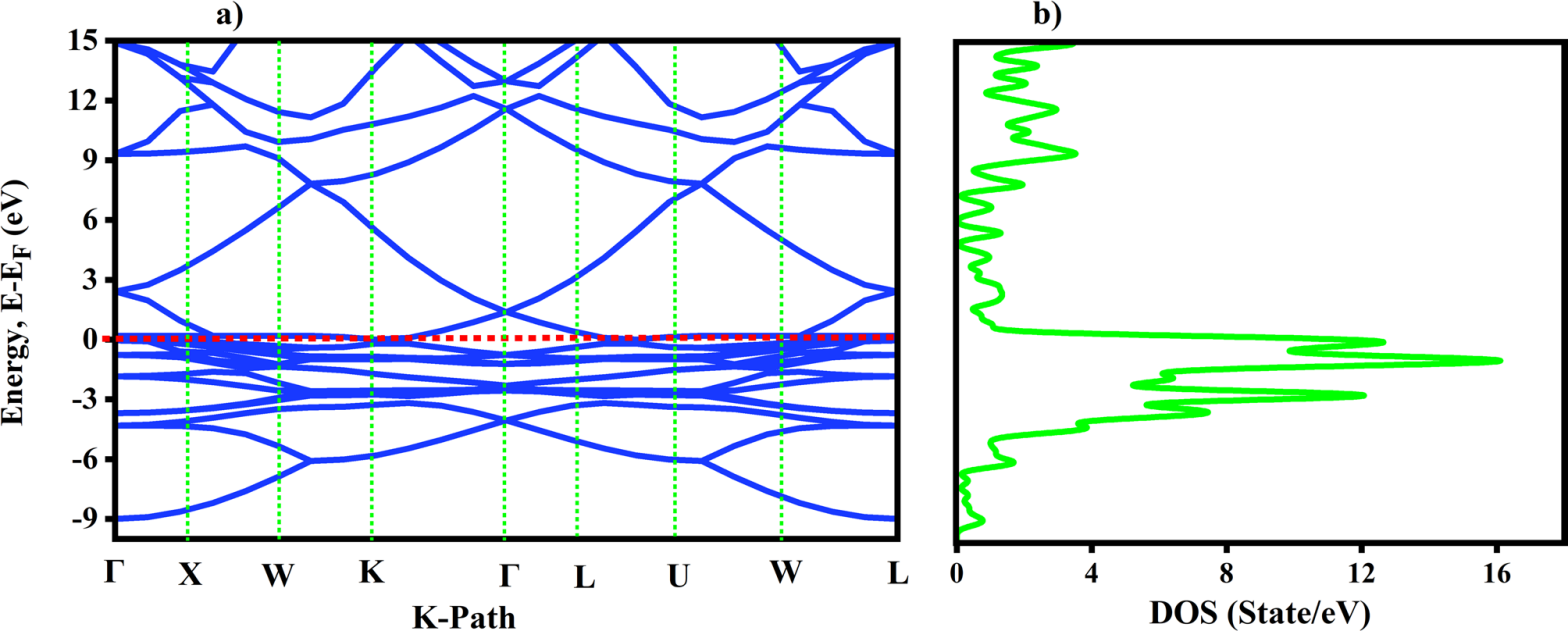
**a**) The electronic band structures of Ni. **b**) DOS of Ni.

However, for Ni the band structure displays a comparatively very small band overlap between the conduction and valence bands as shown in Fig. 6, suggesting that metals have the ability for electrons to flow freely in the conduction band.

### Optical properties of TiO2 and Ni

**Fig. 7:**
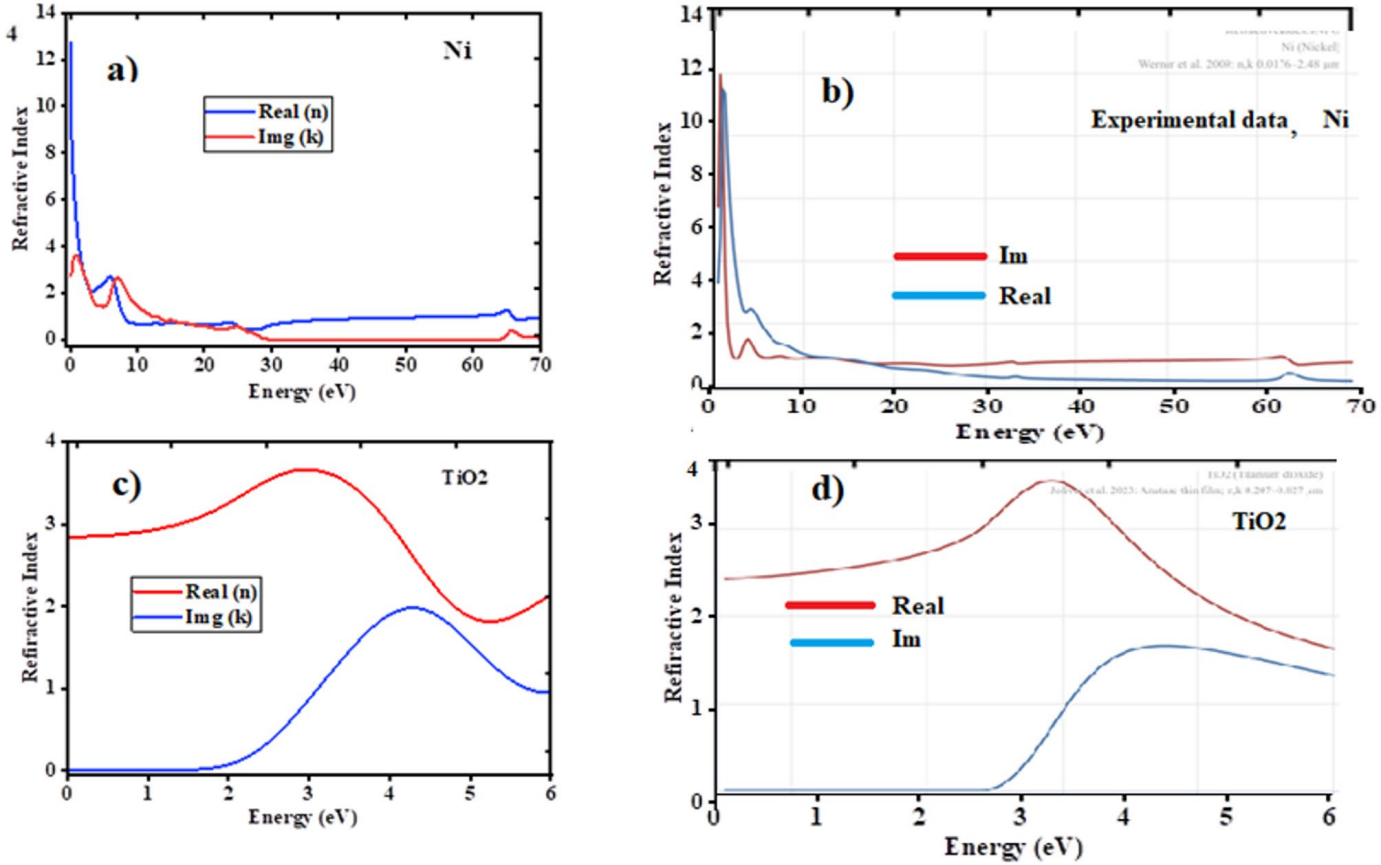
**a**) Refractive index of Ni by DFT calculated. **b**) Experimental refractive index of Ni from database. **c**) Refractive index of TiO2 by DFT calculated. **d**) Experimental refractive index of TiO2 from database.

The electronic transitions within the crystal were taken into consideration when analyzing the optical characteristics of TiO2 and Ni crystal species. When an electron within the crystal interacts with an electromagnetic wave, it leads to the excitation of electrons from the ground state to higher energy states. The real and imaginary parts of the refractive in were calculated along different polarization directions. The polarization direction considered in this work is [100] for both TiO2 and Ni. To compare the DFT values with the experimental data, the imaginary and real parts of the refractive index for the crystal structure of Ni were calculated in the energy range of 0–70 eV, as shown in Fig. 7 (a) and (b). However, for the crystal structure of TiO2, the imaginary and real parts of the refractive index were calculated in the energy range of 0–6 eV, as shown in Fig. 7 (c) and (d). These results agree with the experimental study for Ni^43^ and the theoretical study for TiO2 (2023). Additionally, the experimental data were taken from a database, as shown in Fig. 7 (b) and Fig. 7(d) for Ni and TiO2, respectively.

The refractive index provides insight into how light propagates through a material, which impacts its optical absorption. The refractive index analysis shows that TiO2 crystal has the highest refractive index of 2.9 for the [100] directions and Ni crystal has 12 for the [100] direction, which could enhance interactions between light and the materials, improving photo absorptions and efficacy in selective emitter applications.

**Fig. 8:**
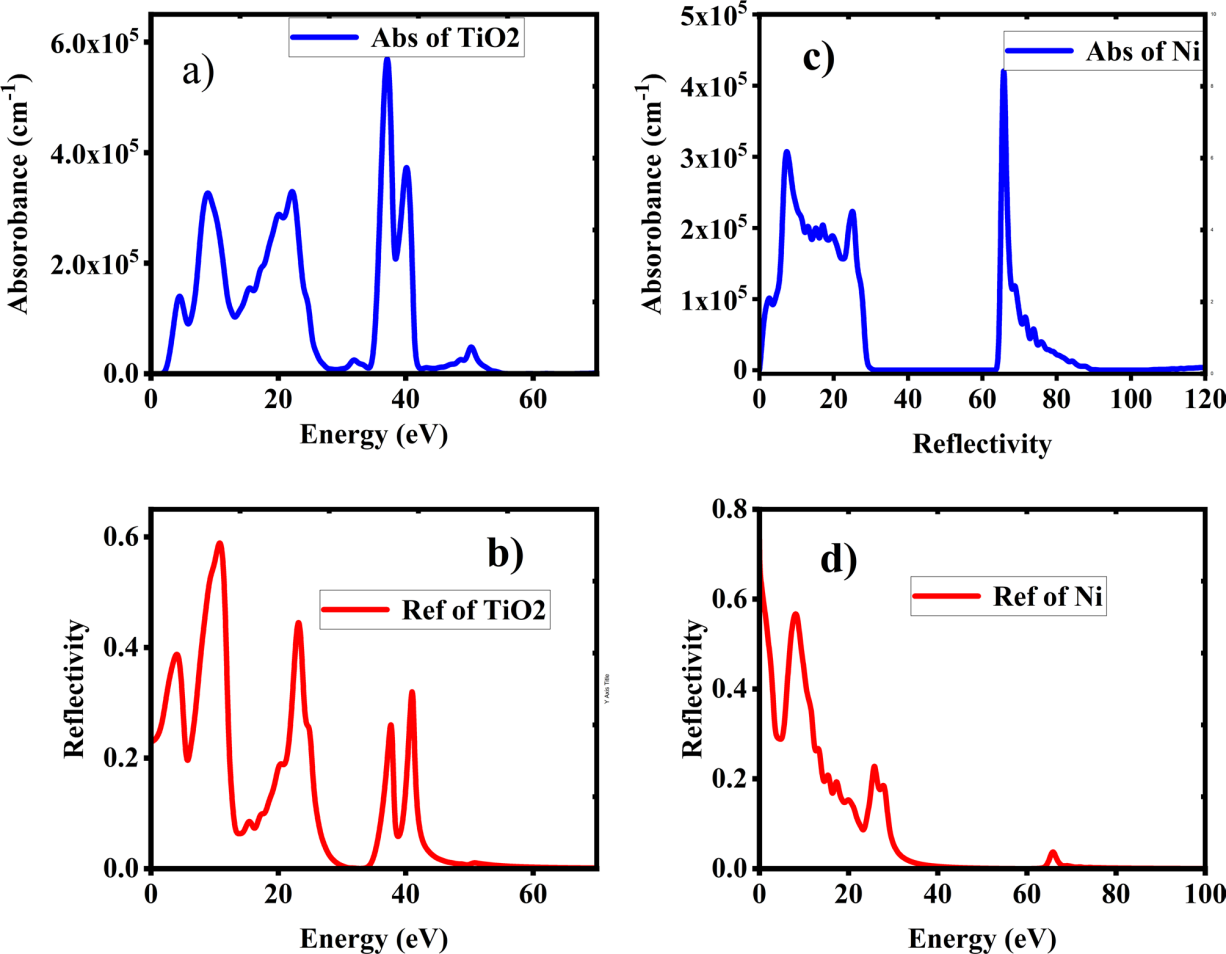
**a**) Absorbance of TiO2. **b**) Reflectivity of TiO2. **c**) Absorbance of Ni. **d**) Reflectivity of Ni.

Furthermore, we have also analyzed the absorption and the reflectivity of TiO2 and Ni crystals. Therefore, the analysis suggests that TiO2 and Ni demonstrates the most promising optical properties for selective emitter applications due to its high static dielectric constant and refractive index. Titanium dioxide (TiO2) and nickel (Ni) each have unique absorption and reflection properties due to their distinct electronic and structural characteristics as shown in Fig. 8.

TiO2 is a wide-bandgap semiconductor with a bandgap around 3.0 eV. This allows it to absorb ultraviolet (UV) light effectively as shown in Fig. 8 (a), as photons with energy greater than the bandgap excite electrons from the valence band to the conduction band. TiO2 also reflects visible light efficiently as shown in Fig. 8 (b) due to its high refractive index, which causes scattering and reflection when light interacts with its surface. These properties make TiO2 ideal for uses such as selective absorber and photo catalysis. In contrast, nickel is a metal with free electrons that contribute to its high reflectivity across the visible spectrum as shown in Fig. 8 (d). The conduction electrons in Ni resonate with incoming light, reflecting it rather than absorbing it. However, nickel does absorb certain wavelengths, especially in the infrared range as shown in Fig. 8 (c), due to interactions between the electromagnetic waves and the electron density in the metal. This combination of reflection and absorption makes nickel valuable in applications where both thermal and optical properties are important.

Optimization is a crucial approach for improving impedance matching, which enhances surface absorbance efficiency within atmospheric transparency windows. The energy density of electromagnetic waves passing through materials is affected by both electric and magnetic fields. The designed absorber/emitter achieves impedance matching with free space^44,45^ by aligning the structure’s impedance with that of the incoming signal through optimization, thereby maximizing the absorption or transmission of solar irradiation. During the simulation, the effects of various parameters on the absorber/emitter’s performance are evaluated. The emitter reached maximum selective and ultra-broadband emissivity across all atmospheric windows, optimized at $\:{\text{h}}_{1}=3{\upmu\:}\text{m},\:\:{\text{h}}_{2}=2.5\:{\upmu\:}\text{m},\:\:{\text{h}}_{3}=0.3\:{\upmu\:}\text{m},\:{\text{h}}_{4}=0.5\:{\upmu\:}\text{m},\:{\text{h}}_{5}=3{\upmu\:}\text{m},\:\:\text{a}=0.15{\upmu\:}\text{m},\:\text{L}=13{\upmu\:}\text{m}\text{b}=0.7{\upmu\:}\text{m},\:\:,\:\text{a}\text{n}\text{d}\:\text{P}=16{\upmu\:}\text{m}$, as illustrated in Fig. 4(b) (blue line).

**Fig. 9:**
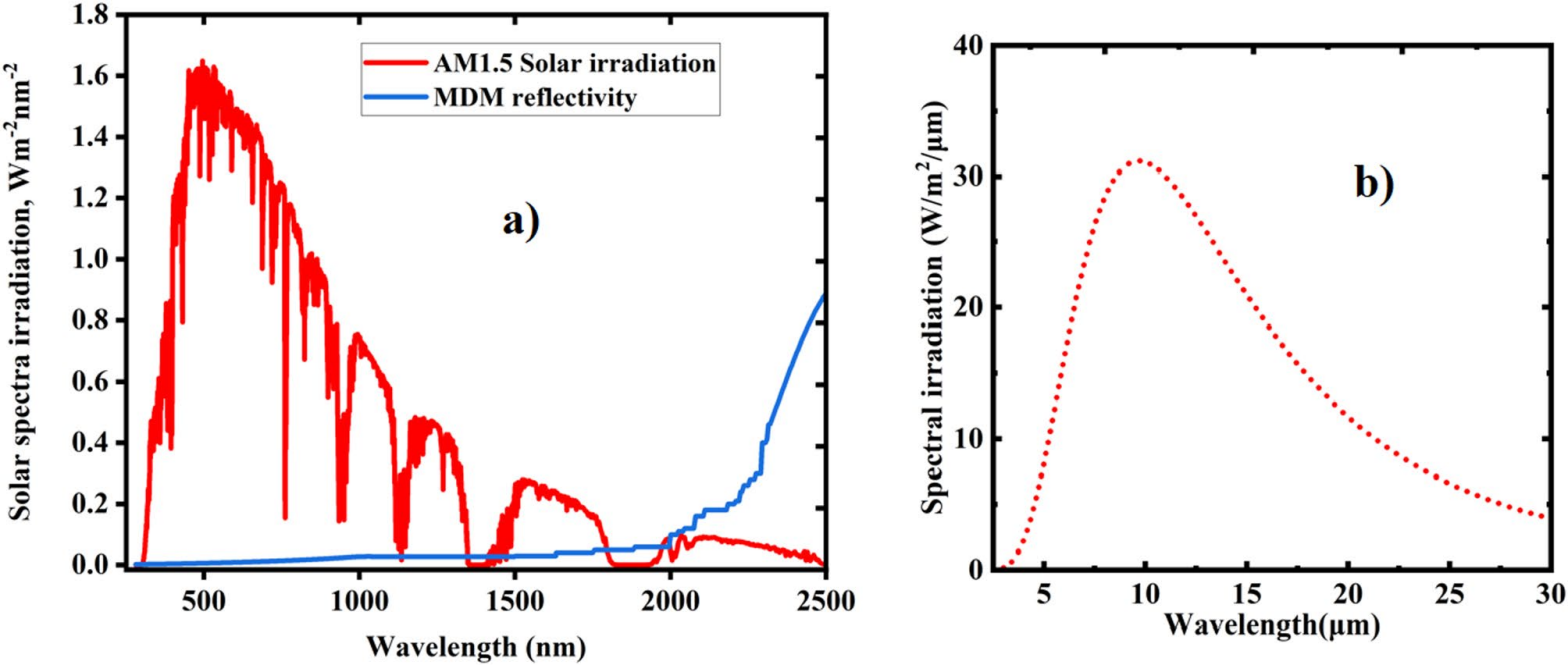
(**a**) A reference of AM 1.5 global solar spectra (red area) and emissivity of designed MDM cooler (blue line). (**b**) The blackbody radiation.

**Fig. 10:**
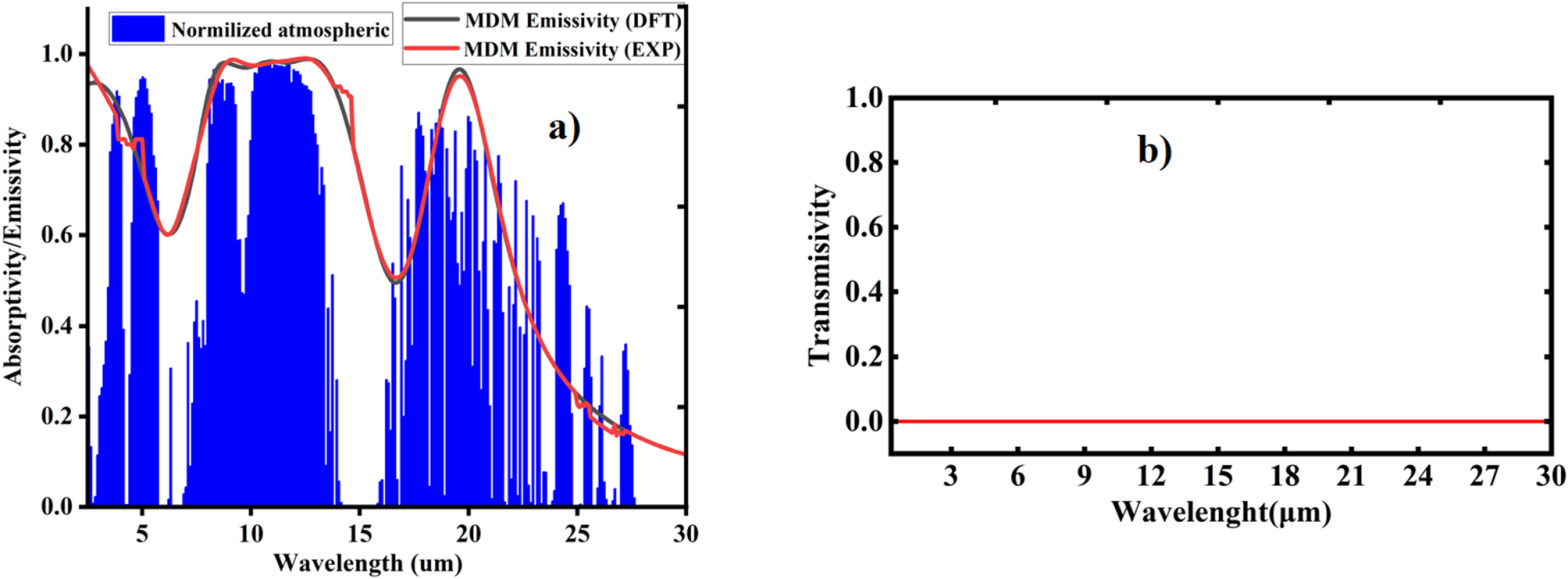
(**a**) A modeled normalization of infrared atmospheric transmittance (blue area) and simulated emissivity for experimental data (red line) and DFT data (black line). (**b**) Transmittance of cooler.

Figure 9 (a) shows the global AM1.5 solar irradiance spectrum (red area) for wavelengths below 2.5 μm, along with the emitter’s reflectivity (blue line). Since the reflectance of solar radiation at wavelengths greater than 2.5 μm has a negligible impact on net cooling^5^, our analysis concentrated on the 0.3 μm to 2.5 μm range to evaluate the structure’s reflection of incident solar energy, $\:{\text{P}}_{\text{s}\text{u}\text{n}}$, as illustrated in Fig. 4 (a) (blue line). We also considered the three atmospheric windows$\:(2.5\mu {\rm{m}} - 5\:\mu {\rm{m}},\:8\:\mu {\rm{m}} - 13\mu {\rm{m}},\:{\rm{and}}\:16\mu {\rm{m}} - 27\mu {\rm{m}})$, placing particular emphasis on the latter two, as they correspond to the regions of higher thermal radiation from a blackbody at approximately 300 K, as shown in Fig. 10 (a). The direct normal solar irradiation ($\:{\text{P}}_{\text{s}\text{u}\text{n}}$) was calculated using $\:{I_{AM1.5}}\left( \lambda \right) = \:994\:W/{m^2}$.

Enhancing overall net cooling power presents key challenges, primarily in increasing mean emissivity within the target windows and improving mean reflectivity of solar radiation. The peak solar irradiation intensity occurs in the 0.3 μm to 1.5 μm wavelength range, where the designed cooler achieves a mean solar reflectance of 98.5% at both optimized and normal incidence angles. With optimized parameters$\:{\text{h}}_{1}=3{\upmu\:}\text{m},\:{\text{h}}_{2}=2.5\:{\upmu\:}\text{m},\:{\text{h}}_{3}=0.3\:{\upmu\:}\text{m},\:\:\text{b}=0.7\:{\upmu\:}\text{m},\:\:\text{h}5=3{\upmu\:}\text{m},\:\:\text{a}\text{n}\text{d}\:\text{P}=16\:{\upmu\:}\text{m}$, the cooler can reflect up to 97% across the entire 0.3 μm to 2.5 μm spectrum, as illustrated in Fig. 9(a) (blue line). This total solar reflectance of 97% is a crucial element in enhancing the performance of net cooling ($\:{\text{P}}_{\text{n}\text{e}\text{t}}$) during the day. Figure 9(b) shows the blackbody radiation and its behavior in the mid-infrared (MIR) region.

This cooler design demonstrates significant emissivity within key atmospheric windows: 0.88 in the 2.5–5 μm range, 0.98 in the 8–13 μm range, and 0.74 in the 16–27 μm range. Achieving such high emittance across the ultraviolet to far-infrared spectrum relies on effective coupling between various resonant modes, including surface plasmon polariton, magnetic polariton, and the tungsten interband transition. The high absorptivity/emissivity observed in the mid-infrared (8–13 μm) is primarily attributed to magnetic polariton resonance. As blackbody radiation is strongest in the MIR region, as illustrated in Fig. 9(b), this contributes significantly to enhancing net cooling power. The final two atmospheric windows are strategically positioned within the blackbody’s peak thermal radiation zone at approximately 300 K. Furthermore, the cooler’s opaque structure exhibits zero transmittance across the entire wavelength range, as shown in Fig. 10(b).

### Angular emissivity and polarization independent

**Fig. 11:**
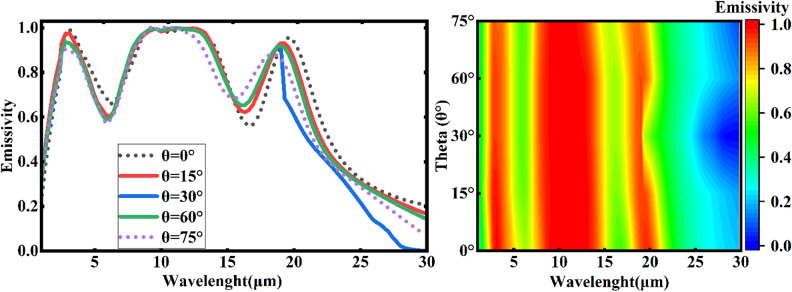
The angle-dependent emissivity of MDM cooler drawn: **a**) by line. **b**) by contour fill.

The impact of incidence angles on the spectral emissivity of MDM emitters is examined, as illustrated in Fig. 11(a). The absorbance/emittance curve remained relatively stable when the incidence angle increased from 0° to 75°, indicating that the designed emitter can effectively emit infrared radiation over a broad range of angles. This capability to maintain consistent performance at various angles is crucial for the stability of its net cooling efficiency. Although the emittance curve showed a notable decline at a 75° incidence angle, it still retained a commendable level of emittance. However, the stability of the MDM’s emittance was compromised when the incidence angle exceeded 75°, aligning with the radiative characteristics typical of most non-conductive surfaces and leading to a reduction in cooling power towards zero^46^. Therefore, it can be concluded that the emitter is suitable for plane waves with incident angles ranging from 0° to 75°. Figure 11(b) indicates the impact of incidence angles on the spectral emissivity of MDM emitters as contour fill. As shown in this figure, the red color represents the range where MDM emits most strongly.

**Fig. 12:**
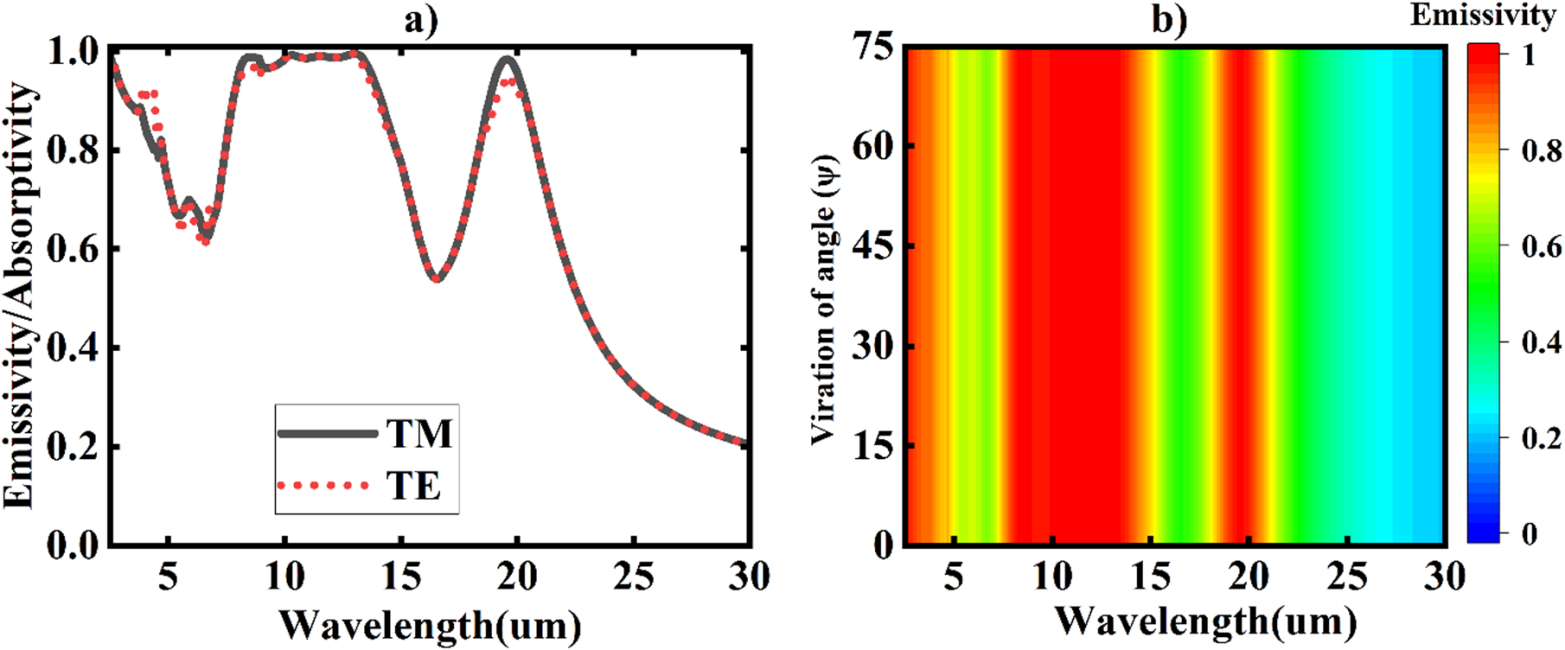
**a**) The computed spectral emittance/absorbance of the cooler for transverse magnetic (TM) polarization is represented by the black line, while the red line indicates transverse electric (TE) polarization at the optimized conditions. (**b**) The spectral absorptivity/emissivity of the optimized MDM cooler is shown as a function of the polarization angle (ψ).

We investigated the polarization independence of the emitter. The spectral emissivity of the MDM emitter was simulated using an electric field vector rotating around the z-axis in the xy plane. As shown in Fig. 12(a), the simulated values for both transverse magnetic (TM) and transverse electric (TE) fields at an incidence angle of 0° exhibit nearly complete overlap. Conversely, Fig. 12(b) demonstrates that the performance of the designed emitter remains consistent as the azimuthal angle (ψ) increases from 0° to 75°. This finding indicates that the designed absorber/emitter is polarization-independent due to its rotational asymmetry. Furthermore, even at an incidence angle of 0°, the symmetry of the designed emitter is evident.

**Fig. 13:**
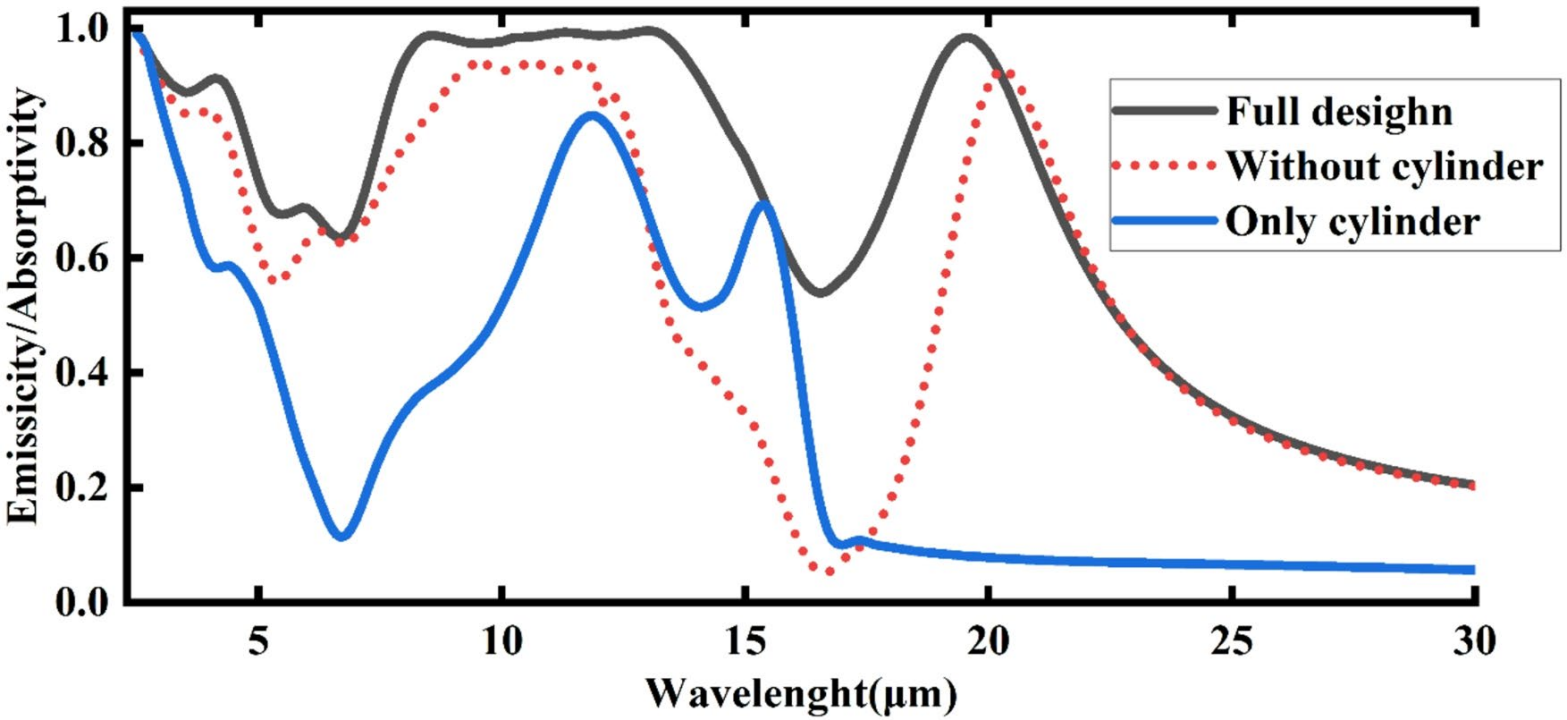
Desighned of three different MDM cooler strucure: Full (black), without cylander(red) and only cylinder(blue).

Figure 13 illustrates the absorbance/emittance performance of three different cooler structures: the honeycomb complete, optimized design (black line), a design lacking cylinders (red line), and a design without the honeycomb structure (blue line). The results show that the widest band of emittance is achieved by the cooler that incorporates both the cylindrical and honeycomb configurations (full design). The whole honeycomb design exhibits the best results, with modest absorption outside of the atmospheric windows (2.5–5 μm, 8–13 μm, and around 16–27 μm) and significant peaks inside. Significant radiative cooling is ensured by this selective behavior, which minimizes undesired heating from broadband absorption and maximizes heat release in the atmospheric windows.

Conversely, the cylinder-free form shows comparatively high emissivity over a broad wavelength range, including outside the intended atmospheric windows. It still has some cooling capacity, but its efficiency is diminished by its lack of precise selectivity. In contrast to the full design, its broadband absorption reduces net cooling performance by retaining more thermal energy rather than radiating it away. The cylinder-only design performs the worst, with emissivity values typically falling small in the majority of crucial ranges. A few little peaks do show up, but they are insufficiently powerful to effectively chill the area. This suggests that the cylinder by itself is unable to attain the required spectrum selectivity and that its function is only successful when paired with the other structural elements of the entire design.

### Performance of passive radiative cooling

The primary objective of this article is to assess the performance of passive radiative cooling. To determine the total radiative cooling efficiency, Kirchhoff’s law helps establish the average absorbance of the designed cooler, which is then used to compute the overall net cooling performance as described in Eqs. 5–9. The radiative cooling efficiency of a cooler is significantly influenced by various factors, including the power emitted from the cooler’s surface, the heat absorbed from the atmosphere, and non-radiative heat received from the surrounding environment, as well as the cooler’s solar reflectance profile. Based on solar irradiation, net cooling efficiency is categorized into two main types: daytime and nighttime efficiency. The net cooling power, as a function of surface temperature $\:\left({\text{T}}_{\text{s}\text{u}\text{r}}\right)$, is calculated without considering convection and conduction heat transfer.

**Fig. 14:**
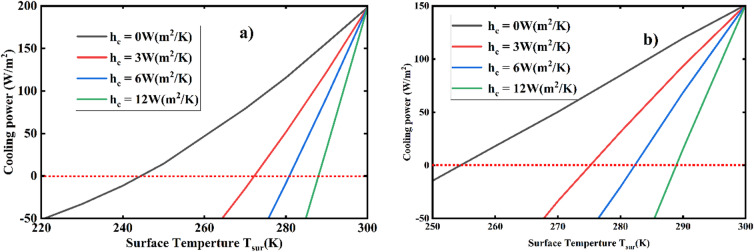
**a**) Total cooling power ($\:{\text{P}}_{\text{n}\text{e}\text{t}}$) as a function of surface temperature during nighttime ($\:{\text{T}}_{\text{a}\text{m}\text{b}}$ = 300 K) for various heat transfer coefficients (hc). (**b**) Total cooling power ($\:{\text{P}}_{\text{n}\text{e}\text{t}}$) as a function of surface temperature during daytime ($\:{\text{T}}_{\text{a}\text{m}\text{b}}$ = 300 K) for different heat transfer coefficients (hc).

Additionally, the net cooling efficiency at night was assessed both with and without factoring in non-radiative heat exchange, as illustrated in Fig. 14(a). Since sunlight is unavailable at night, the cooling efficiency improves as the surface emissivity increases until the surface temperature equals the ambient temperature ($\:{\text{T}}_{\text{s}\text{u}\text{r}}$ - $\:{\text{T}}_{\text{a}\text{m}\text{b}}$ = 0). This phenomenon occurs because the surface maintains a consistent emissivity, allowing it to selectively absorb energy from the environment to balance the energy it emits. Consequently, the cooler achieved a net cooling power of 198 W/m² at an ambient temperature with a steady-state temperature of 244 K, enabling a reduction of 56 K below the assumed ambient temperature, as shown by the black line in Fig. 14(a). While many researchers have reported effective net cooling methods during the night, the results of this study are notably high and impressive.

To assess the statistics of this research, the relationship between emissivity and wavelength under various conditions was analyzed using a correlation coefficient (R²). This coefficient reflects the quality of the plotted graphs, and based on the polynomial function, R² was calculated for all graphs. The resulting correlation coefficients for this study range from 0.9 to 0.98, with the exception of the graph depicting net cooling power versus surface temperature for both daytime and nighttime. Nonetheless, the primary finding of our study is the high net cooling power achieved. The correlation coefficients for surface temperature and net cooling were calculated separately using a linear function for both daytime and nighttime, as presented in Table 1. As indicated in Table 1, the observed correlation coefficients are 0.987 < R² < 0.999 during the day and 0.988 < R² < 0.97 at night. This result suggests that the plotted graphs are well correlated.

**Table 1:** Correlation coefficient of surface temperature versus net cooling power at different conduction or convection coefficients during daytime and nighttime.

No	At Day time	At Night time
	Correlation coefficient ($\:{\mathbf{R}}^{2}$)	Correlation coefficient ($\:{\mathbf{R}}^{2}$)
$\:{\rm{hc}}\: = \:0\:({\rm{W}}/({{\rm{m}}^2}\:{\rm{K}})$	0.97	0.989
$\:{\rm{hc}}\: = \:3\:({\rm{W}}/({{\rm{m}}^2}\:{\rm{K}})$	0.98	0.99
$\:{\rm{hc}}\: = \:6\:({\rm{W}}/({{\rm{m}}^2}\:{\rm{K}})$	0.99	0.98
$\:{\rm{hc}}\: = \:6\:({\rm{W}}/({{\rm{m}}^2}\:{\rm{K}})$	0.99	0.99

Furthermore, conduction and convection heat exchange are considered to evaluate the cooler’s performance at night. The cooling power decreases as the conduction and convection coefficients increase, particularly when the surface temperature is lower than the assumed ambient temperature. The steady-state temperature at night was calculated using heat transfer coefficients of 3, 6, and 12 (W/(m² K)), as shown in Fig. 14 (red, blue, and green). The designed cooler achieved temperatures below the ambient temperature by 29 K, 20 K, and 13 K, respectively. This result indicates that the designed MDM emitter demonstrates high net cooling performance at night, thanks to its elevated average emissivity in the targeted atmospheric windows, while sunlight is absent.

While achieving high cooling efficiency during the day is challenging, this research demonstrated that the MDM cooler attained a net cooling power of 150.4 W/m² at ambient temperature under direct solar irradiation. This allowed the cooler to reach a thermal equilibrium temperature of 256 K, indicating that it can lower the ambient temperature by 44 K, excluding the effects of parasitic convection and conduction, as illustrated in Fig. 14(b) (black line). This result reflects a high efficiency compared to previous studies, such as those by Raman et al.^20^ and Lin et al.^13^.

Moreover, the efficiency of the cooler is further evaluated by incorporating the effects of conduction and convection, as illustrated in Fig. 14(b) (red, blue, and green lines). Even when accounting for non-radiative heat exchange, the cooler can still achieve temperatures below the ambient level. To accurately assess the impact of non-radiative heat exchange, three different combined conduction and convection coefficients, hc = 3, 6, and 12 (W/(m² K)) were considered. With an ambient temperature of 300 K, the calculated steady-state temperatures for hc = 3, 6, and 12 (W/(m² K)) are 273.5 K, 281 K, and 288 K, respectively. Given that the optimal surface temperature is 300 K, the designed cooler reached temperatures below ambient by 26.5 K, 19 K, and 12 K for hc = 3, 6, and 12 (W/(m² K)), respectively. This result demonstrates that even with high conduction and convection coefficients (hc = 12 (W/(m² K))) used for net cooling power calculations, the cooler can still effectively lower temperatures below ambient during the daytime.

Conversely, we evaluated the emittance performance of each MDM structure shown in Fig. 13, highlighting the significant role that the MDM structure plays in ultra-broadband selective emitters. Different structural designs yield varying spectral efficiencies, with the cooler achieving net cooling powers of 150.4 W/m² for the full design, 101.3 W/m² for the design without the cylinder, and 40.6 W/m² for the design featuring only the cylinder (without the honeycomb). To validate the cooling performance of these structures, we considered non-radiative heat exchange. For instance, with conduction and convection coefficients of 12 W/(m² K), the designed cooler reached temperatures below ambient by 10.2 K, 8 K, and 3.4 K for the full design, only the honeycomb (without the cylinder), and only the cylinder (without the honeycomb), respectively. Overall, Table 2 analyzes the net cooling power for the full design, the design without the cylinder, and the metal-dielectric-metal structure with only the cylinder.

**Table 2:** Comparison of the three designed cooler structures during the daytime.

Parameters	Full MDM designed	Only honeycomb MDM designed	Only cylinder MDM designed
Net cooling power ($\:{\mathbf{P}}_{\mathbf{n}\mathbf{e}\mathbf{t}}$)	$\:150.4\:{\bf{W}}/{{\bf{m}}^2}\:$	101.3 $\:{\bf{W}}/{{\bf{m}}^2}\:$	40.6 $\:{\bf{W}}/{{\bf{m}}^2}\:$
$\:\text{S}\text{t}\text{e}\text{a}\text{d}\text{y}-\:\text{t}\text{e}\text{m}\text{p}\text{e}\text{r}\text{a}\text{t}\text{u}\text{r}\text{e}\:\text{a}\text{t}\:\text{h}\text{c}\:=$	256 K	269 K	283.4 K
Cool down below ambient temperature; $\:{\rm{At}}\:{\rm{hc}}\: = \:0\:({\rm{W}}/({{\rm{m}}^2}\:{\rm{K}}))\:$	44 K	31 K	14.6 K
At $\:{\rm{hc}}\: = \:3\:({\rm{W}}/({{\rm{m}}^2}\:{\rm{K}}))\:$	26 K	19.4 K	9 K
At $\:{\rm{hc}}\: = \:6\:({\rm{W}}/({{\rm{m}}^2}\:{\rm{K}}))\:$	20 K	14.7 K	6.6 K
At $\:{\rm{hc}} = 12\:({\rm{W}}/({{\rm{m}}^2}\:{\rm{K}}))$	14.3 K	10 K	4.3 K

**Fig. 15:**
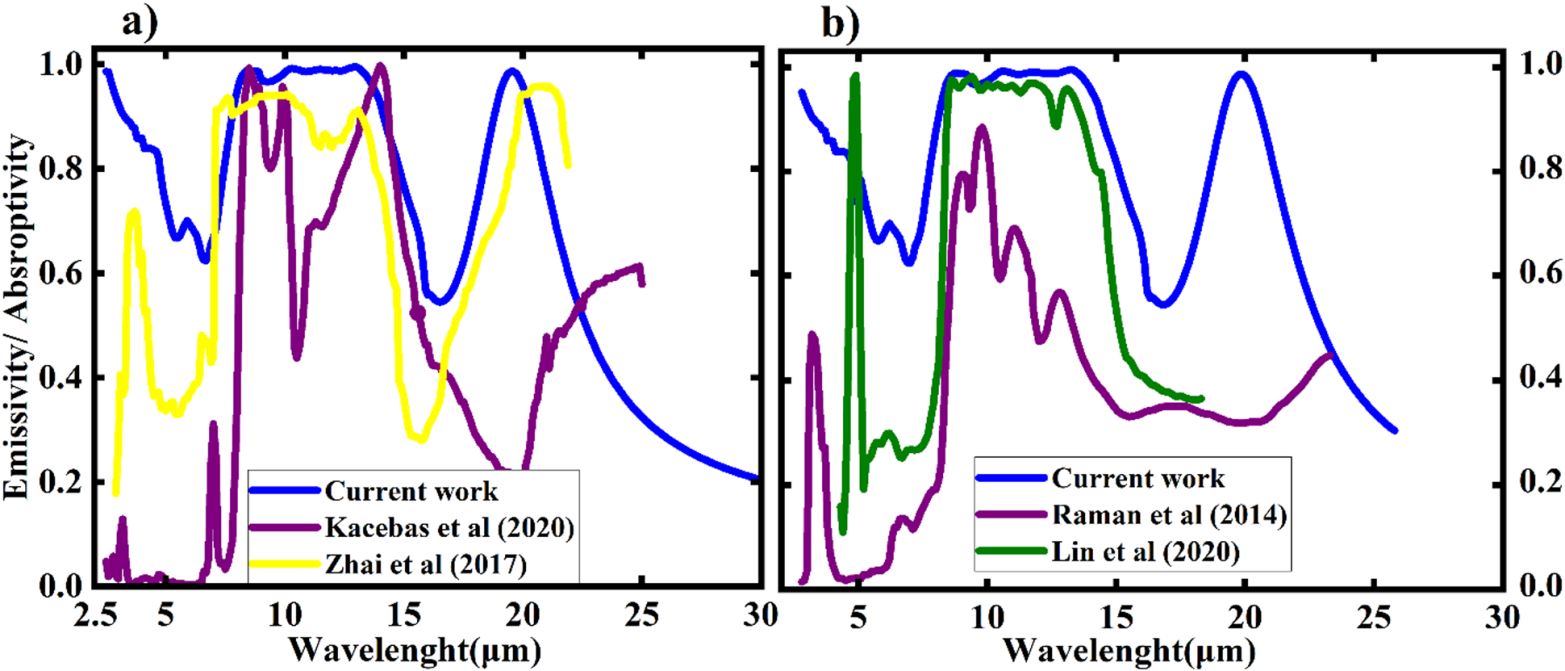
**a**) Emissivity of MDM as reported by Kecebas et al. (red line) and Zhai et al. (blue line). **b**) Emissivity of photonic materials as reported by Raman et al. (red line) and Lin et al. (blue line).

Moreover, we referenced previously reported numerical and experimental studies on metamaterial and photonic structures to compare and illustrate our results. For instance, the work of Zhai et al. (2017)^14^ and Kecebas et al. (2020)^28^ investigated hybrid MDM structures, as shown in Fig. 14(a) (black dotted line) and MDM with seven layers, respectively, depicted in Fig. 15(a) (red line). Additionally, Raman^20^ developed a photonic thin film with ten layers, achieving a net cooling power of 40.1 W/m², which can cool down to 32 K (5 °C) below ambient temperature, as illustrated in Fig. 15(b) (red line). In contrast, at 994 W/m² of solar radiation, the 3-layer TiO₂–Ni metal–dielectric–metal structure used in this work achieved a noticeably higher net cooling power of 150.2 W/m². The high net cooling power and increased infrared emission range suggest greater radiative cooling performance compared to the earlier designs, even though the temperature reduction for the current study is not stated directly. Table 3 provides a clear summary of our current work alongside previous studies, focusing on structure, number of layers, types of materials, AM1.5 solar irradiation, cooling performance below ambient temperature, and net cooling power. This information is clearly outlined in Table 3. Our cooler simplifies the structure and reduces fabrication difficulties compared to the multi-layer photonic structures of Raman et al. (2014) and Kecebas et al. (2020), while maximizing cooling performance relative to the work of Lin et al. (2022) and Zhai et al. (2017).

**Table 3:** Comparation of cooler performance current study with privous studies.

Name of scholars	Structure	Layers	Matrials	Infraread Wavelength ranges	AM1.5 Solar-irradiation	Ambiant temp	Net cooling power	Cool below amb temp
Zhai et al.^14^	MM	Hybrid	SiO_2_,Ag	2.5–25 μm	900 Wm^− 2^	300 K	93 Wm^− 2^	TDX
Raman et al.^20^	photonic	10	SiO_2_, HfO_2_,Ti, Si	2.5–25 μm	850 Wm^− 2^	300 K	40.1Wm^− 2^	32 K (5 ${}^ \circ {\rm{C)}}$
Kacebas et al.^28^	photonic	9	TiO_2_, SiO_2_,Ag, Si	2.5–25 μm	850 Wm^− 2^	300 K	100 Wm^− 2^	40 K
Curent study	MDM	3	TiO_2_, Ni	2.5–27 μm	994 Wm^− 2^	300 K	150.4 Wm^− 2^	44

### Model verification

**Fig. 16:**
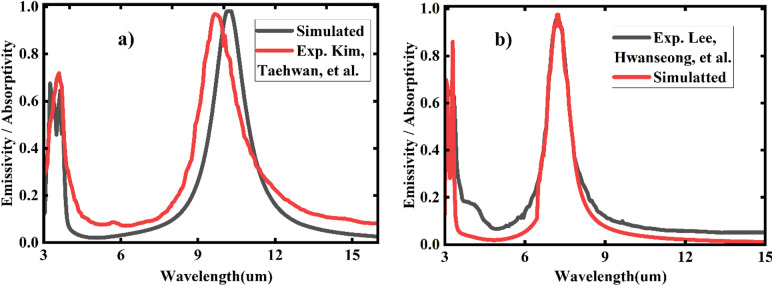
(**a**)Experimental (red line) and simulated (black line) absorptivity/emissivity of emitter (**b**) Experimental (back line) and simulated (red line) absorptivity/emissivity of emitter.

To ensure the model’s validity, the data’s authenticity was verified using multiple cross-validation methods. Initially, simulation results were compared against prior experimental studies by Lee et al.^7^ and Kim et al.^29^, as illustrated in Figs. 16(a) and 16(b), respectively. Specifically, Fig. 16(a) compares the simulated emissivity (black line) of a cylindrically designed metal-dielectric-metal (MDM) emitter with experimental emissivity data (red line) from Kim et al., using a cylinder with a 1.8 μm diameter, 3 μm unit cells, and a 0.2 μm dielectric thickness. Similarly, Fig. 16(b) presents a comparison between the simulated emissivity (red line) and the experimental results (black line) reported by Lee et al., employing a cylinder with a 1.57 μm diameter, 3 μm unit cells, and a 0.1 μm dielectric thickness. The strong agreement between the numerical results obtained via the Finite Element Method (FEM) in COMSOL and the experimental data confirms the reliability of the FEM method for generating accurate data.

**Fig. 17:**
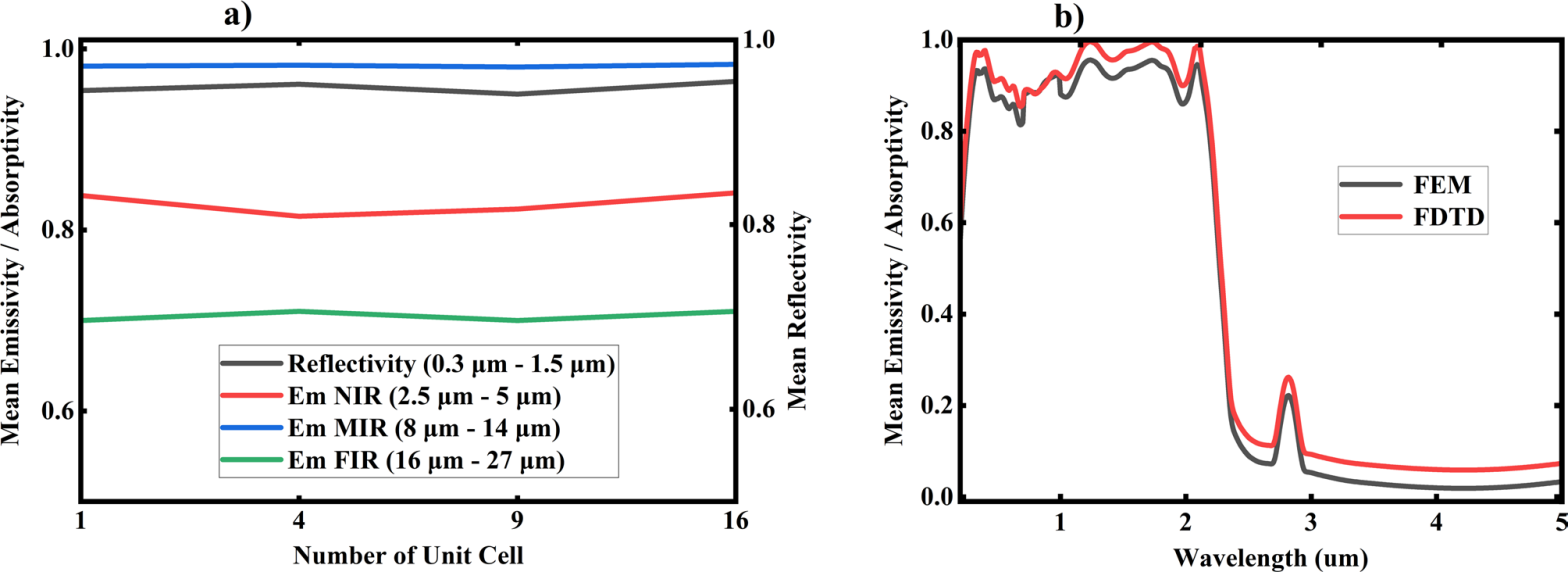
Simulation of emitter with: **a**) different number of unit cell **b**) FEM anf FDTD method.

In this study, we investigated how the number of unit cells affects both emittance and reflectance properties to validate the data. As shown in Fig. 17 (a), the number of unit cells ranged from 1 to 16, with only slight variations in the average reflectance and emittance as the number of unit cells increased. These results confirm that the FEM method is a dependable tool for generating precise data. When choosing the mesh size, it is crucial to find a balance between computational accuracy and valid outcomes. Given that the simulated spectra for solar reflection and thermal emissivity stabilize at smaller mesh sizes, we selected a maximum mesh size of 0.3 μm to ensure the accuracy of the simulation results. Additionally, in 2021, Chen et al.^25^ demonstrated the effectiveness of the FEM tool in their study on microsphere coatings for passive cooling, utilizing both FEM and FDTD methods also we compared to validate as shown on Fig. 17 (b).

## Conclusion

The use of spectrally emissive structures designed for the optical properties of materials has a variety of applications. A broadband and selective emitter functioning within the infrared atmospheric transparency window can greatly improve radiative cooling efficiency. In this study, we designed a metal-dielectric-metal (MDM) resonator specifically for radiative cooling. Measurements of absorptivity indicated a wide-angle absorption/emission that closely corresponds to the primary infrared atmospheric transparency window. The cooler achieved emissivity values of 0.88 in the first window (2.5–5 μm), 0.985 in the middle window (8–13 μm), and 0.74 in the last window (16–27 μm). Additionally, it can reflect up to 0.97% across the entire wavelength range of 0.3–2.5 μm. The emitter exhibited excellent radiative cooling performance, reaching a net cooling power of 150.4 W/m² during the day and 198 W/m² at night. This cooling power enables temperature drops of 44 K during the day and 56 K at night below ambient levels. Overall, the proposed cooler/emitter structure offers a promising approach for passive radiative cooling, as it reduces peak atmospheric cooling demand, provides high net cooling power, uses cost-effective materials, is easy to manufacture, and minimizes greenhouse gas emissions.

## Data Availability

The data used to support the findings of this study are available from the corresponding author upon request.
